# Physiological Metabolic Responses of *Ophraella communa* to High Temperature Stress

**DOI:** 10.3389/fphys.2019.01053

**Published:** 2019-08-27

**Authors:** Hongsong Chen, Ghulam Sarwar Solangi, Chenchen Zhao, Lang Yang, Jianying Guo, Fanghao Wan, Zhongshi Zhou

**Affiliations:** ^1^State Key Laboratory for Biology of Plant Diseases and Insect Pests, Institute of Plant Protection, Chinese Academy of Agricultural Sciences, Beijing, China; ^2^Guangxi Key Laboratory for Biology of Crop Diseases and Insect Pests, Institute of Plant Protection, Guangxi Academy of Agricultural Sciences, Nanning, China; ^3^Department of Entomology, Sindh Agriculture University Sub Campus, Umerkot, Pakistan; ^4^State Key Laboratory of Cotton Biology, Institute of Cotton Research, Chinese Academy of Agricultural Sciences, Henan, China

**Keywords:** leaf beetle, heat stress, developmental stage, physiological responses, common ragweed

## Abstract

Considering the predicted rising temperatures under current climate change and heat wave scenarios, organisms are expected to suffer more intense and frequent thermal stress. Induced heat is accumulated by organisms and can cause a variety of physiological stress responses. *Ophraella communa* is an effective biological control agent of common ragweed, *Ambrosia artemisiifolia*, but the responses of this biocontrol agent to heat stress have not been fully elucidated and, therefore, its potential responses to climate change are uncertain. We investigated the physiological metabolism of subsequent *O. communa* adults after: (1) different developmental stages (egg, larval, pupal, and adult) were exposed to thermal stress for 3 h each day for 3, 5, 5, and 5 days, respectively (by stage); and (2) individuals were exposed to thermal stress throughout the egg-to-adult period for 3 h each day. The high temperatures of 40, 42, and 44°C were used to induce thermal stress. A control group was reared at 28 ± 2°C. The results showed that short- or long-term exposure to daily phasic high temperatures significantly decreased water and lipid contents and significantly increased glycogen and glycerol contents in all adults (i.e., after exposure of different stages or throughout the egg-to-adult period). However, the total sugar content significantly increased in adults after the eggs and larvae were exposed to brief short-term thermal stress. Compared to the control, the total sugar content was also significantly higher in the adults and pupae exposed to 44°C. Total sugar content in females increased significantly in response to long-term phasic thermal stress at 40°C. However, sugar content of males exposed to 44°C decreased significantly. After long-term phasic thermal stress, water and glycogen contents in males were significantly higher than in females; however, females had higher total sugar and lipid contents. Therefore, our study provides a basic understanding of the metabolic responses of *O. communa* to thermal stress and offers insights into its potential as a natural biocontrol agent against *A. artemisiifolia* during the summer season and under predicted climate change scenarios.

## Introduction

Not only is the common ragweed, *Ambrosia artemisiifolia* L. (Asterales: Asteraceae, hereafter referred to as ragweed) a highly invasive alien species that causes substantial crop-yield losses in many parts of the world, but its prolific production of highly allergenic pollen also creates considerable public health problems (Essl et al., 2015; Zhou et al., 2017). In China, this weed was unintentionally introduced into the southeastern coastal region in the 1930s (Wan and Wang, 1988), and can now be found in 21 provinces (Zhou et al., 2017). The ragweed leaf beetle, *Ophraella communa* LeSage (Coleoptera: Chrysomelidae) is indigenous to North America, and has been shown to be an effective biological control agent of ragweed (Zhou et al., 2017). *Ophraella communa* larvae and adults preferentially feed on both the leaves and meristems of ragweed (Cardarelli et al., 2018), and when the population pressure is sufficiently high, plants are completely defoliated, and flowering and seed set of most plants are prevented (Müller-Schärer et al., 2014; Zhou et al., 2014). Although some plants do manage to survive until reproduction, their seed production has been shown to be significantly reduced (Throop, 2005; Zhou et al., 2014).

*Ophraella communa* was first recorded in China in 2001 (Meng and Li, 2005), where it was shown to be an effective biocontrol agent against ragweed (Zhou et al., 2014, 2017; Chen et al., 2018a). In the presence of *O. communa*, the spread of ragweed in China has been greatly suppressed by reduced flowering, seed set, and dispersal (Zhou et al., 2017). The *O. communa* beetles have also been successful in reducing ragweed densities in crop fields in Canada (Teshler et al., 2002), and the effectiveness of this agent against ragweed in Europe is currently under evaluation (Cardarelli et al., 2018). A decrease in airborne pollen, allergenic potential, and medical costs of ragweed was observed in the areas of Italy and France where *O. communa* was released (Bonini et al., 2018; Cardarelli et al., 2018; Mouttet et al., 2018).

Temperature is arguably the most important abiotic factor that influences insect physiology, behavior, and fitness (Hance et al., 2007). Climate change and global warming have resulted in an increase in the average temperatures and frequency of climatic extremes (e.g., heat waves) worldwide (Rahmstorf and Coumou, 2011; Intergovernmental Panel on Climate Change [IPCC], 2014). The frequency and intensity of heat waves observed in China has increased significantly over the past 50 years (Sun et al., 2014; Wang W.W. et al., 2016; Kang and Eltahir, 2018), particularly since the beginning of the twenty-first century (Sun et al., 2014; Kang and Eltahir, 2018). As ectotherms, insects exposed to extreme temperatures may suffer irreversible losses of fitness or even death (Dillon et al., 2009). Many insects have complex life cycles, and different life stages (ages/instars) may experience different seasonal environments, inhabit different habitats and microclimates, and thus, experience different environmental stressors. They also have different levels of physiological sensitivity and may respond differently (Coyne et al., 1983; Kingsolver et al., 2011). Therefore, the negative effects of extreme temperatures on insects depend, in part, on the life stage that experiences the heat stress (Chiu et al., 2015). However, insects do exhibit physiological and behavioral responses to thermal stress that must be regulated by proper thermosensation mechanisms (Kim et al., 2015). There is a dearth of data available on physiological responses to temperature and other climatic factors across life stages (Tucić, 1979). The ability of ectotherms to flexibly alter physiological mechanisms in response to changes in environmental temperatures (plasticity/acclimation/acclimatization) determines their capacity to maintain their performance and fitness despite environmental variation (Kern et al., 2015). Esperk et al. (2016) showed that populations can cope with elevated heat stress by evolving a higher basal heat tolerance (evolutionary response) or stronger induced heat tolerance (plastic response), or both. Recent studies have demonstrated examples of the adaptive acclimation responses of insects to thermal stress through changes in their metabolome profiles (Hariharan et al., 2014; Schou et al., 2017). These plastic responses are proposed to be important for insect fitness in thermally variable environments (Bahrndorff et al., 2009). Polyols and heat shock proteins are expressed in response to cold and heat shock, and that pretreatment at high temperatures increases tolerance of cold shock (Sejerkilde et al., 2003; Sinclair and Chown, 2003). Depending on the species, the responses to heat and cold may be similar. Insects accumulate sugar alcohols (polyols) to protect cell membranes and protein stability (Salvucci, 2000). Lipid reserves are also important for insects to meet their energy demand during diapause (Hahn and Denlinger, 2007), provide energy for the developing embryo (Ziegler and Van Antwerpen, 2006), and to fuel prolonged periods of flight (Beenakkers et al., 1984). Glycerol has been recognized as a chemical chaperone and may act synergistically with heat shock proteins to generate cryoprotection (Chowanski et al., 2015). Unsaturation of membranes is a well-documented response to thermal stress and polyols, and to prevent membrane fluid-to-gel transitions (Logue et al., 2000; Hochachka and Somero, 2002; Hulbert, 2003).

The optimum developmental temperatures for the *O. communa* life cycle in the laboratory was shown to range from 25 to 28°C, and the survival of the first-instar larvae gradually decreased at ambient temperatures >28°C (Zhou et al., 2010). At 36°C, there was 100% mortality of the first-instar larvae and the survival of other instars and the female fecundity decreased significantly (Zhou et al., 2010). High summer temperatures of over 40°C are not rare in central and southern China^1^. The maximum recorded temperature was 44.1°C (Gong et al., 2014), and extreme high-temperature events are expected to increase in these regions (Chen S.S. et al., 2018). Owing to climate change, we predict that high summer temperatures of 40–44°C will be increasingly common in future heat waves. High temperatures are inevitable during the summertime in central and southern China, and populations of *O. communa* have been reported to decrease during the summer season in these regions (Zhou et al., 2011; Chen et al., 2014). Our previous studies indicated that the development, survival, fecundity, and adult body size of *O. communa* were adversely affected after the different developmental stages were exposed to high temperatures (≥40°C) for brief periods (Zhou et al., 2011; Chen et al., 2014, 2018c). In China, *O*. *communa* will inevitably encounter high temperature stress in the field in summer, during the vigorous growing stage of ragweed.

Thermal stress in one life stage can affect fitness in later stages in ectotherms with complex life cycles (Zhang et al., 2015; Zheng et al., 2017; Chen et al., 2018c). To our knowledge, many studies have focused on the effects of extreme events on the treated life stage but seldom involve carry-over effects on subsequent stages. The adult life stage is the most important for distribution and reproduction, and determines the future development of the population. Our previous study demonstrated that the fecundity of *O*. *communa* adults significantly changed when eggs, larvae, pupae, and adults were exposed to high temperatures (Chen et al., 2018c). Therefore, research on the adult physiological responses in *O*. *communa* affected by heat stress in different developmental stages is necessary. In the present study, we investigated the physiological responses of *O*. *communa* to heat stress. Considering the scenarios of extreme summer temperatures in southern China, the objectives of present studies focused on assessing the effects of thermal stress on the physiological metabolism of reproductively mature *O*. *communa* adults and their changes in water, total sugar, lipid, glycogen, and glycerol content after (1) the different development stages (egg, larval, pupal, and adult) were exposed to brief, short-term high temperature treatments or (2) the entire egg-to-adult period was exposed to brief, long-term high temperature treatments. Understanding the physiological responses of *O*. *communa* to thermal stress is essential to accurately predict the impacts of climate change on the distribution, population phenology, and ultimately the biological control efficacy of this species.

## Materials and Methods

### Host Plants

*Ambrosia artemisiifolia* seeds were collected, stored, and sown as described in our previous studies (Chen et al., 2018b, c). When seedlings reached a height of ∼15 cm, the apical buds were removed to prevent apical dominance. The seedlings were transplanted into pots (21 × 17 cm, diameter × height; one seedling per pot) that contained vegetable garden soil. A total of 500 ragweed seedlings were prepared. All plants were watered daily and fertilizer was applied (N:P:K = 13:7:15) twice per month to maintain growth (Zhou et al., 2010). When the plants were ∼50 cm high, ∼400 of the potted plants were used for *O. communa* eggs, larvae, pupae, adults, and egg-to-adult period heat treatments (Chen et al., 2018b, c).

### Insect Culture

The collecting and rearing of *O. communa* adults were conducted as described in our previous studies (Chen et al., 2018b, c). Adult pairs (*n* = 30) of *O. communa* were randomly collected from the reared colony, and each individual was transferred with the aid of a fine brush (size 0) onto a fresh, potted ragweed plant, which was then covered with nylon gauze (40 mesh size). After allowing 1 or 2 days for oviposition, the adult beetles were removed and the plants that housed newly laid eggs were placed in the greenhouse (temperature: 28 ± 2°C; natural light conditions; relative humidity: 70 ± 5%) for normal growth until the desired life stage was achieved, e.g., eggs ≤ 12 h old, first-instar larvae ≤ 24 h old, pupae ≤ 24 h old, adults ≤ 12 h old (Chen et al., 2018b, c).

### Heat Treatment Intensities and Durations

The heat treatment intensities and durations used in the present study were as described in our previous studies (Zhou et al., 2010; Chen et al., 2014, 2018b, c). Briefly, we selected 40, 42, and 44°C for 3 h per day and the treatment of 28°C was used as the control. The exposure periods for the eggs, larvae, pupae, and adults were 3, 5, 5, and 5 days, respectively. The exposure periods of the egg-to-adult treatments were 25–30 days based on the developmental velocity at each temperature. The high-temperature exposure treatments were performed separately in environmental chambers (PRX-450D, Ningbo Haishu Safe Experimental Equipment Co., Ltd., Zhejiang, China) with a maximum error of 1°C, a relative humidity of 70 ± 5%, a photoperiod of 14:10 (L:D) h, and a light intensity of 12,000 lx. Each treatment was replicated three times.

### Short-Term Phasic Thermal Stress on Eggs, Larvae, Pupae, and Adults of *O. commun*a

Eggs ≤ 12 h old (*n* = 100), first-instar larvae ≤ 24 h old (*n* = 120), pupae ≤ 24 h old (*n* = 30) were separately placed on three potted plants in the greenhouse at 28 ± 2°C under natural light conditions. Fifteen ragweed plants were randomly selected for each developmental stage (i.e., 15 plants × 3 treatments × 3 developmental stages) and for the control (15 plants × 3 developmental stages). Therefore, a total of 180 ragweed plants were used. The ragweed plants with the eggs, larval, and pupal developmental stages were then exposed to high temperatures for 3 h daily for 3, 5, and 5 consecutive days, respectively (by stage) in environmental chambers (Zhou et al., 2010; Chen et al., 2014, 2018b, c).

Following high temperature stress, treated pupae were collected by detaching the leaves they were on and transferring them to transparent plastic boxes (19 × 12 × 6 cm) that were maintained in an unsealed cuvette plastic tube covered with nylon gauze (60 mesh size) in the laboratory at 28 ± 2°C and 70 ± 5% relative humidity. Pupae were checked daily for adult emergence. The treated eggs and larvae were maintained in the greenhouse until they reached the pupal stage. The process for these pupae was the same as that for the treated pupae following high temperature stress. The sex of each newly emerged adult was visually determined (Guo et al., 2010). The adults were maintained in the laboratory at 28 ± 2°C and 70 ± 5% relative humidity for 5 days (Zhou et al., 2010; Chen et al., 2014, 2018b, c).

Newly emerged adults ≤ 12 h old (*n* = 600 pairs) were randomly selected from the greenhouse culture for phasic high-temperature exposure treatments. Adults were sexed and 50 pairs were placed on a flourishing potted ragweed plant (50 cm height) in a cage (40 × 40 × 60 cm), which constituted one replicate. The ragweed plants that contained the adults were exposed to high temperatures in environmental chambers for 3 h daily for 5 consecutive days, after which the infested potted plants were maintained in a greenhouse, the ragweed plants were changed daily, and a total of 60 ragweed plants was used (Zhou et al., 2010; Chen et al., 2014, 2018b, c).

### Long-Term Phasic Thermal Stress on *O. commun*a

Approximately 1000 eggs ≤ 12 h old were retained on each potted plant in the greenhouse. Fifteen such plants were selected for exposure to each high temperature treatment in environmental chambers for 3 h daily until the emergence of adults. The infested potted plants were maintained in a greenhouse, and a total of 60 ragweed plants were used. Two hundred pairs of newly emerged adults ≤ 12 h old were placed on one potted strong and fresh ragweed plant (50 cm height) in a cage (40 × 40 × 60 cm) and covered with nylon gauze (60 mesh size); this constituted one replicate. The *O*. *communa* adults on ragweed plants were then exposed to high temperatures in the environmental chambers for 3 h daily for 5 consecutive days, after which the infested potted plants were maintained in a greenhouse, the ragweed plants were changed daily, and a total of 60 ragweed plants were used (Zhou et al., 2010; Chen et al., 2014, 2018b, c).

### Water, Total Sugar, Lipid, Glycogen, and Glycerol Content Assays

After exposure to high temperatures, the water, total sugar, lipid, glycogen, and glycerol content was determined in subsequent adult females and males. Water content analyses were replicated 20 times, while total sugar, lipid, glycogen, and glycerol content analyses were replicated three times.

### Water

Alive female (*n* = 20) and male (*n* = 20) adults were rapidly frozen using liquid nitrogen, and weighed individually on a precision electronic balance (Make and Model sensitivity 0.1 mg) to determine their fresh weight (FW). Similarly, dry weight (DW) was calculated after drying for 48 h at 60°C. Water content (WC,%) was calculated as follows (Hua et al., 2014):

(1) $$\text{WC}=\frac{\text{FW}-\text{DW}}{\text{FW}}\times 100$$

### Total Sugar and Glycogen

Alive female (*n* = 30) and male (*n* = 30) adults were rapidly frozen using liquid nitrogen, and then were carefully brushed to remove contaminating particles, weighed, and homogenized in 200 μL of 2% Na_2_SO_4_. An additional 1300 μL chloroform–methanol (1:2) was added to the homogenate to extract the simple carbohydrates of the adults. Individual homogenates were centrifuged for 10 min at 7150 × *g*. To determine the amount of carbohydrates in the adults, a sample of the supernatant (300 μL) was mixed with 200 μL distilled water and reacted for 10 min at 90°C with 1 mL of anthrone reagent (500 mg anthrone dissolved in 500 mL concentrated H_2_SO_4_). Absorbances were measured at 630 nm using a spectrophotometer. The concentration of the component was determined from a standard curve by using glucose (Sigma) as the standard (Bemani et al., 2012).

Glycogen content was determined from the centrifugation pellet which was washed in 400 μL of 80% methanol to remove the possible remnants of sugar. To extract the glycogen, 250 μL distilled water was added to the washed pellet, and the mixture was heated for 5 min at 70°C. Subsequently, 200 μL of the solution was removed and reacted for 10 min at 90°C with 1 mL anthrone reagent (600 mg anthrone dissolved in 300 mL concentrated H_2_SO_4_). The optical density was read at 630 nm using a spectrophotometer. The amount of glycogen in the sample was determined from a standard curve by using glycogen (Sigma) as the standard (Bemani et al., 2012).

### Lipid and Glycerol

Collected female (*n* = 120) and male (*n* = 120) adults were stored at −20°C until lipid content was measured. Specimens were dried at 60°C for 24 h and weighed (DW_1_). Lipids were extracted from the dried adults by putting 40 specimens into a 60 mL Soxhlet extractor with petroleum ether as medium for 24 h at 40°C. This step was repeated twice, after which the lipid extract was stored at −20°C for glycerol content measurement. The specimens were dried again at 60°C for 24 h and weighed (DW_2_). The lipid content (LC,%) was calculated as follows (Hua et al., 2014):

(2) $$\mathrm{LC}=\frac{{\text{DW}}_{1}-{\text{DW}}_{2}}{{\text{DW}}_{1}}\times 100$$

Glycerol was measured using the cupric glycerinate colorimetry method. The lipid extract was transferred to a 10 mL volumetric flask, diluted with petroleum ether to volume, and heated to 80°C for 15 min. The liquid was then centrifuged at 8500 × *g* for 10 min after which 1 mL volume of the supernatant was added to a 10 mL centrifuge tube containing 3.8 mL of saturated Cu(OH)_2_ solution (0.3 mL 150 mg/mL CuSO_4_ + 3.5 mL 50 mg/mL NaOH), and mixed for 5 min. The centrifuge tube was then centrifuged at 5000 × *g* for 10 min. The concentration of cupric glycerinate was measured at 630 nm. The glycerol content in each sample was determined from a standard curve by using glycerol (Sigma) as the standard. Sterile-distilled water was used as the control (Hua et al., 2014).

### Statistical Analyses

All data were analyzed using SPSS 21.0 (SPSS Inc., Chicago, Illinois, United States) and were checked for normality and homoscedasticity before performing ANOVA. Data were analyzed by Tukey’s HSD (honestly significant difference) test (one-way ANOVA) when they met the normal distribution and homogeneity of variance at 0.05 level, otherwise by Kruskal-Wallis one-way ANOVA (*k* samples) (independent samples, non-parametric tests) (McDonald, 2009). *P*-values < 0.05 indicated significant differences and data were reported as means ± SE (standard error of the mean) (Chen et al., 2018b, c).

## Results

### Water Content

Overall, the water content in both subsequent *O. communa* male and female adults decreased after the different developmental stages were exposed to brief thermal stress treatments, compared to the control. Stage-specific differences were observed in the females when the different developmental stages were exposed to 40 and 44°C. Stage-specific differences were also observed in males when the different developmental stages were exposed to 40 and 42°C (Tables 1, 2).

**TABLE 1:** Mean (± SE) water content of *Ophraella communa* female adults when eggs, larvae, pupae, and adults were exposed to thermal stress, i.e., 28 (control), 40, 42, and 44°C for 3 h each day for 3, 5, 5, and 5 days, respectively (by stage).

**Temperature (°C)**	**Water content (% fresh weight)**			
	**Eggs**	**Larvae**	**Pupae**	**Adults**
28	68.9 ± 0.6ab, A	69.7 ± 0.8a, A	69.9 ± 0.8a, A	70.5 ± 0.6a, A
40	70.9 ± 0.6a, A	68.4 ± 0.6a, B	67.5 ± 0.4ab, B	67.2 ± 0.4b, B
42	67.0 ± 0.5b, A	65.1 ± 0.7b, A	65.5 ± 0.5b, A	67.0 ± 1.8ab, A
44	64.6 ± 0.7c, B	65.6 ± 0.5b, B	70.2 ± 0.7a, A	64.0 ± 1.6b, B

*Values within the same column followed by different lowercase or uppercase letters within the same row are statistically different (Tukey’s HSD or Kruskal-Wallis test, *P* < 0.05).*

**TABLE 2:** Mean (± SE) water content of *Ophraella communa* male adults when eggs, larvae, pupae, and adults were exposed to thermal stress, i.e., 28 (control), 40, 42, and 44°C, for 3 h each day for 3, 5, 5, and 5 days, respectively (by stage).

**Temperature (°C)**	**Water content (% fresh weight)**			
	**Eggs**	**Larvae**	**Pupae**	**Adults**
28	70.3 ± 0.8a, A	70.0 ± 0.8a, A	69.7 ± 0.7a, A	70.5 ± 0.6a, A
40	70.4 ± 0.6a, A	68.8 ± 0.7ab, AB	67.5 ± 0.5ab, B	67.7 ± 0.5b, B
42	67.7 ± 0.6b, A	65.1 ± 0.6c, B	65.7 ± 0.5b, AB	67.0 ± 0.7b, AB
44	69.0 ± 0.5ab, A	67.1 ± 0.6bc, A	68.5 ± 0.5a, A	68.9 ± 0.7ab, A

*Values within the same column followed by different lowercase or uppercase letters within the same row are statistically different (Tukey’s HSD or Kruskal-Wallis test, *P* < 0.05).*

**FIGURE 1:**
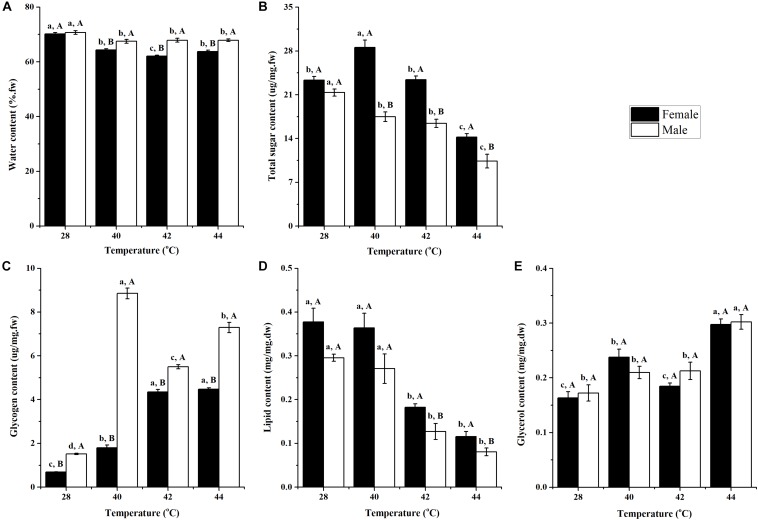
Effect of long-term phasic thermal stress (egg to adult) on water **(A)**, total sugar **(B)**, glycogen **(C)**, lipid **(D)**, and glycerol **(E)** content of *Ophraella communa* adults. The temperature 28°C served as the control. Each value represents the mean (± SE) for adult females and males of three replicates (20 replicates for water content). Key: fw = fresh weight, dw = dry weight. Different lowercase letters indicate a significant difference for the same gender among temperatures (Tukey’s HSD or Kruskal-Wallis test, *P* < 0.05). Different uppercase letters indicate a significant difference between males and females at the same temperature (Tukey’s HSD or Kruskal-Wallis test, *P* < 0.05).

After long-term phasic thermal stress, the water content of *O. communa* female (*F*_3__,__76_ = 62.95, *P* < 0.0001) and male (*P* = 0.0030) adults decreased significantly compared to the control; and at high temperatures, the male adults were observed to have a higher water content than the adult females (Figure 1A).

### Total Sugar Content

After the eggs and larvae were exposed to brief thermal stress treatments, the total sugar content in both subsequent *O. communa* female (eggs: *F*_3__,__8_ = 22.77, *P* = 0.0003; larvae: *F*_3__,__8_ = 97.44, *P* < 0.0001) and male (eggs: *F*_3__,__8_ = 15.57, *P* = 0.0007; larvae: *F*_3__,__8_ = 28.99, *P* = 0.0001) adults increased significantly compared to the control. Except for the 44°C treatment, the total sugar content in both subsequent *O. communa* female and male adults was also significantly higher than the control after the pupae and adults were exposed to high temperatures. Stage-specific differences were observed in females at all high temperatures but only observed in males when the different developmental stages were exposed to 40 and 44°C (Tables 3, 4).

**TABLE 3:** Mean (± SE) total sugar of *Ophraella communa* female adults when eggs, larvae, pupae, and adults were exposed to thermal stress, i.e., 28 (control), 40, 42, and 44°C, for 3 h each day for 3, 5, 5, and 5 days, respectively (by stage).

**Temperature (°C)**	**Total sugar content (ug/mg fresh weight)**			
	**Eggs**	**Larvae**	**Pupae**	**Adults**
28	24.9 ± 0.8c, A	25.1 ± 0.4c, A	27.0 ± 0.9b, A	25.0 ± 0.9c, A
40	30.0 ± 1.3bc, B	36.5 ± 0.5b, AB	43.8 ± 3.1a, A	42.9 ± 1.6a, A
42	33.2 ± 2.3b, B	36.6 ± 0.9b, B	46.5 ± 2.7a, A	37.8 ± 0.9b, B
44	43.3 ± 1.8a, A	44.4 ± 1.1a, A	19.2 ± 1.1b, B	19.4 ± 0.6d, B

*Values within the same column followed by different lowercase or uppercase letters within the same row are statistically different (Tukey’s HSD test, *P* < 0.05).*

**TABLE 4:** Mean (± SE) total sugar of *Ophraella communa* male adults when eggs, larvae, pupae, and adults were exposed to thermal stress, i.e., 28 (control), 40, 42, and 44°C, for 3 h each day for 3, 5, 5, and 5 days, respectively (by stage).

**Temperature (°C)**	**Total sugar content (ug/mg fresh weight)**			
	**Eggs**	**Larvae**	**Pupae**	**Adults**
28	20.8 ± 0.3c, AB	18.6 ± 0.5c, B	22.3 ± 0.5ab, A	21.5 ± 1.1b, A
40	24.8 ± 0.8b, AB	24.0 ± 0.8b, B	21.3 ± 2.7b, B	31.9 ± 1.4a, A
42	26.3 ± 1.1b, A	27.2 ± 1.2b, A	29.8 ± 1.4a, A	28.5 ± 0.6a, A
44	31.5 ± 1.7a, A	32.2 ± 1.5a, A	18.4 ± 1.3b, B	17.5 ± 0.6b, B

*Values within the same column followed by different lowercase or uppercase letters within the same row are statistically different (Tukey’s HSD test, *P* < 0.05).*

After long-term phasic thermal stress, the total sugar content in *O. communa* female adults at 40°C was significantly higher than the control; however, the opposite trend was observed at 44°C. In the *O. communa* male adults, whereby their total sugar content was significantly lower than the control in any of the high temperature treatments. Female adults were observed to have a higher total sugar content than males in all high temperature treatments (Figure 1B).

### Glycogen Content

After the different developmental stages were exposed to brief thermal stress treatments, the glycogen content in both subsequent *O. communa* female (except from the treatments of eggs at 40 and 42°C, larvae at 40 and 44°C, and pupae at 42 and 44°C) and male adults (except from the treatments of eggs at 40 and 42°C, and larvae at all high temperatures) were significantly higher than the control. Stage-specific differences were also observed in both females and males when the different developmental stages were exposed to the high temperature treatments (Tables 5, 6).

**TABLE 5:** Mean (± SE) glycogen content of *Ophraella communa* female adults when eggs, larvae, pupae, and adults were exposed to thermal stress, i.e., 28 (control), 40, 42, and 44°C, for 3 h each day for 3, 5, 5, and 5 days, respectively (by stage).

**Temperature (°C)**	**Glycogen content (ug/mg fresh weight)**			
	**Eggs**	**Larvae**	**Pupae**	**Adults**
28	0.88 ± 0.06b, A	0.69 ± 0.03b, B	0.69 ± 0.01b, B	0.72 ± 0.04d, AB
40	1.04 ± 0.02b, B	1.43 ± 0.02ab, AB	7.86 ± 0.25a, AB	8.62 ± 0.26a, A
42	1.72 ± 0.04b, D	8.42 ± 0.16a, A	7.10 ± 0.08ab, B	5.38 ± 0.29b, C
44	7.34 ± 0.75a, A	5.13 ± 0.07ab, B	3.39 ± 0.11ab, BC	1.76 ± 0.10c, C

*Values within the same column followed by different lowercase or uppercase letters within the same row are statistically different (Tukey’s HSD or Kruskal-Wallis test, *P* < 0.05).*

**TABLE 6:** Mean (± SE) glycogen content of *Ophraella communa* male adults when eggs, larvae, pupae, and adults were exposed to thermal stress, i.e., 28 (control), 40, 42, and 44°C, for 3 h each day for 3, 5, 5, and 5 days, respectively (by stage).

**Temperature (°C)**	**Glycogen content (ug/mg fresh weight)**			
	**Eggs**	**Larvae**	**Pupae**	**Adults**
28	1.64 ± 0.06b, A	1.61 ± 0.09ab, AB	1.48 ± 0.08c, AB	1.23 ± 0.10c, B
40	1.26 ± 0.06b, C	1.07 ± 0.03b, C	4.60 ± 0.33b, B	6.83 ± 0.24a, A
42	1.49 ± 0.04b, C	6.14 ± 0.12a, B	8.56 ± 0.29a, A	7.02 ± 0.34a, B
44	2.64 ± 0.15a, B	2.61 ± 0.00ab, B	4.17 ± 0.22b, A	2.76 ± 0.06b, B

*Values within the same column followed by different lowercase or uppercase letters within the same row are statistically different (Tukey’s HSD or Kruskal-Wallis test, *P* < 0.05).*

The glycogen contents of *O. communa* female and male adults were significantly higher than the control after long-term (egg-to-adult) phasic thermal stress. Higher glycogen contents were observed in male adults than in female adults, both in the high temperature treatments and compared to the control (Figure 1C).

### Lipid Content

After the different developmental stages were exposed to short-term thermal stress, the lipid content in subsequent *O. communa* female (except for every stage at 40°C) and male adults decreased significantly compared to the control. No stage-specific differences were observed in females and males in response to any of the high temperature treatments and compared to the control (Tables 7, 8).

**TABLE 7:** Mean (± SE) lipid content of *Ophraella communa* female adults when eggs, larvae, pupae, and adults were exposed to thermal stress, i.e., 28 (control), 40, 42, and 44°C, for 3 h each day for 3, 5, 5, and 5 days, respectively (by stage).

**Temperature (°C)**	**Lipid content (ug/mg dry weight)**			
	**Eggs**	**Larvae**	**Pupae**	**Adults**
28	0.38 ± 0.03a, A	0.37 ± 0.02a, A	0.38 ± 0.02a, A	0.31 ± 0.01a, A
40	0.37 ± 0.02a, A	0.39 ± 0.02a, A	0.32 ± 0.03ab, A	0.30 ± 0.02ab, A
42	0.29 ± 0.01a, A	0.26 ± 0.01b, A	0.24 ± 0.01bc, A	0.22 ± 0.02bc, A
44	0.28 ± 0.03a, A	0.27 ± 0.03b, A	0.21 ± 0.02c, A	0.20 ± 0.02c, A

*Values within the same column followed by different lowercase or uppercase letters within the same row are statistically different (Tukey’s HSD test, *P* < 0.05).*

**TABLE 8:** Mean (± SE) lipid content of *Ophraella communa* male adults when eggs, larvae, pupae, and adults were exposed to thermal stress, i.e., 28 (control), 40, 42, and 44°C, for 3 h each day for 3, 5, 5, and 5 days, respectively (by stage).

**Temperature (°C)**	**Lipid content (ug/mg dry weight)**			
	**Eggs**	**Larvae**	**Pupae**	**Adults**
28	0.27 ± 0.02a, A	0.26 ± 0.03a, A	0.23 ± 0.01a, A	0.23 ± 0.01a, A
40	0.21 ± 0.02ab, A	0.19 ± 0.01ab, A	0.18 ± 0.02ab, A	0.16 ± 0.01b, A
42	0.16 ± 0.01b, A	0.17 ± 0.01b, A	0.15 ± 0.01bc, A	0.12 ± 0.01b, A
44	0.17 ± 0.02b, A	0.16 ± 0.02b, A	0.13 ± 0.02b, A	0.12 ± 0.02b, A

*Values within the same column followed by different lowercase or uppercase letters within the same row are statistically different (Tukey’s HSD test, *P* < 0.05).*

After long-term phasic thermal stress, lipid content in *O. communa* female and male adults decreased, especially at 42 and 44°C. The lipid content in *O. communa* female adults was higher than in the males at all high temperature treatments, and the differences were significant at 42 and 44°C (Figure 1D).

### Glycerol Content

In total, the glycerol content of both the subsequent *O. communa* female and male adults were higher compared to the control, and stage-specific differences were only observed in males when the different developmental stages were exposed to the different high temperature treatments (Tables 9, 10).

**TABLE 9:** Mean (± SE) glycerol content of *Ophraella communa* female adults when eggs, larvae, pupae, and adults were exposed to thermal stress, i.e., 28 (control), 40, 42, and 44°C, for 3 h each day for 3, 5, 5, and 5 days, respectively (by stage).

**Temperature (°C)**	**Glycerol content (mg/mg dry weight)**			
	**Eggs**	**Larvae**	**Pupae**	**Adults**
28	0.17 ± 0.01b, A	0.17 ± 0.01a, A	0.16 ± 0.01a, A	0.17 ± 0.01a, A
40	0.23 ± 0.01a, A	0.21 ± 0.01a, A	0.21 ± 0.01a, A	0.19 ± 0.01a, A
42	0.17 ± 0.01b, A	0.17 ± 0.01a, A	0.19 ± 0.02a, A	0.24 ± 0.02a, A
44	0.19 ± 0.01ab, A	0.21 ± 0.01a, A	0.18 ± 0.01a, A	0.19 ± 0.01a, A

*Values within the same column followed by different lowercase or uppercase letters within the same row are statistically different (Tukey’s HSD test, *P* < 0.05).*

**TABLE 10:** Mean (± SE) glycerol content of *Ophraella communa* male adults when eggs, larvae, pupae, and adults were exposed to thermal stress, i.e., 28 (control), 40, 42, and 44°C, for 3 h each day for 3, 5, 5, and 5 days, respectively (by stage).

**Temperature (°C)**	**Glycerol content (ug/mg dry weight)**			
	**Eggs**	**Larvae**	**Pupae**	**Adults**
28	0.17 ± 0.01c, A	0.18 ± 0.01b, A	0.17 ± 0.01c, A	0.18 ± 0.01ab, A
40	0.29 ± 0.01a, A	0.27 ± 0.01a, A	0.24 ± 0.01b, A	0.15 ± 0.02b, B
42	0.17 ± 0.01c, C	0.18 ± 0.01b, C	0.30 ± 0.01a, A	0.24 ± 0.01a, B
44	0.24 ± 0.01b, A	0.27 ± 0.01a, A	0.18 ± 0.01c, AB	0.22 ± 0.01a, B

*Values within the same column followed by different lowercase or uppercase letters within the same row are statistically different (Tukey’s HSD test, *P* < 0.05).*

After long-term phasic thermal stress, glycerol content in *O. communa* female and male adults increased, especially at 44°C. No significant differences were observed between female and males at high temperatures and the control temperature (28°C) (Figure 1E).

## Discussion

Ectothermic species live in variable environments and must face substantial challenges, including biotic and abiotic stresses, for their survival and reproduction. Temperature is one of the most important abiotic variables that affects ectothermic species. Invertebrates have evolved a variety of responses (i.e., physiological and behavioral) to avoid and withstand variable temperatures, e.g., behavioral activities (seeking shelter) and biophysical actions (changing the fluidity of cell membranes and accumulating sugars). When temperature exceeds the optimum temperature range, there are two mutually exclusive outcomes: survival or death (Denlinger and Yocum, 1998). Even if an individual survives exposure to heat stress, its fitness may subsequently be affected (Wang S.Y. et al., 2016). We previously studied the effects of brief exposure to high temperatures on the life history parameters (Zhou et al., 2011; Chen et al., 2018c) and body size (Chen et al., 2014) of *O*. *communa*, and the antioxidant responses of this beetle to thermal stress (Chen et al., 2018b). Physiological metabolites, such as water (Ju et al., 2014), lipids (Qian et al., 2017), sugars, and polyols (Colinet et al., 2015) reportedly play important roles in organismal responses to thermal stress. Membrane lipid composition, sugar concentration, and polyol concentration can also affect the temperature resistance of insects (Hoffmann et al., 2003). It is also widely acknowledged that physiological thermal tolerance differs depending on life stage (Marais et al., 2009; Blanckenhorn et al., 2014; Terblanche et al., 2017). According to the Bogert effect principle, behavioral thermoregulation allows ectothermic animals to escape lethal temperatures, and less-mobile life stages are expected to show greater plasticity of thermal tolerance than more-mobile life stages (Terblanche et al., 2017). However, little is known about the metabolic responses of *O*. *communa* to high temperature stress, especially in the juvenile stages, which are more likely to be exposed to extreme temperatures in the field (e.g., adults can behaviorally avoid thermal stress but the pre-adult stages, especially the eggs and pupae, cannot) (Hoffmann et al., 2003). In this study, we explored short- and long-term phasic thermal stress-induced effects on water, total sugar, glycogen, lipid, and glycerol contents of *O*. *communa* adults after different life-stages or entire egg-to-adult period were exposed to high temperature stress. Our results indicated that these parameters were significantly altered under both short- and long-term phasic heat stress.

Regulation of water balance in insects is critically important for the maintenance of homeostasis (Phillips and Hanrahan, 1986). Maintaining water balance is a major challenge for insects, and dehydration may lead to significant physiological injury including protein denaturation, nucleic acid damage, and lipid peroxidation, and can ultimately result in death (Lopez-Martinez et al., 2009). Most water loss under ambient temperatures occurs through the cuticle (Hadley, 1994). Cuticular hydrocarbons (CHCs) cover the cuticles of almost all insects, serving as a waterproofing agent and facilitating communication signals (Menzel et al., 2017). At a critical temperature, the CHCs can melt and water loss through the CHC layer increases (Menzel et al., 2017). Thus, melting temperature is directly associated with the ability of an insect to maintain its water balance, i.e., allowing them to withstand high temperatures (Menzel et al., 2017; Duarte et al., 2019). The melting point depends on the chemical composition of the cuticle, and the composition-related differences may be affected by, e.g., life stage and temperature (Duarte et al., 2019). High environmental temperatures create several challenges for ectotherms, including greater water loss through evaporation (Tomlinson and Phillips, 2012). Evaporative water loss of the ichneumonid wasp *Lissopimpla excelsa* showed a significant exponential increase between 12 and 40°C and increased significantly and rapidly between 30 and 35°C (Tomlinson and Phillips, 2012). Similar results were reported for other insects, such as the tenebrionid beetle *Eleodes armata* between 30 and 35°C (Ahearn and Hadley, 1969), and the grasshopper *Melanoplus sanguinipes* between 25 and 35°C (Rourke, 2000). After the exposure of different developmental stages or throughout the egg-to-adult period to brief short- or long-term high temperatures, although most of the subsequent male and female adults water content was significantly lower than that at the control temperature (28°C), the water content did not significantly decrease. We speculate that the compounds of the *O*. *communa* cuticle are rich in longer chains and linear alkanes for waterproofing (Duarte et al., 2019), and that the melting temperature for *O*. *communa* cuticle is greater than 44°C. It has also been reported that body temperature can be regulated through increasing evaporative cooling at high temperatures (Tomlinson and Phillips, 2012). The use of evaporative water loss has been identified as an alternate thermoregulatory strategy in many insects (Prange, 1996), whereby insects can enhance their tolerance to temperature extremes by excreting body water (Chown et al., 2011); therefore, water content in insects always decreases with an increase in temperature. In this study, the *O*. *communa* adult body water content decreased significantly after the different developmental stages were exposed to short- or long-term brief thermal stress treatments or when the specimens were exposed to these treatments during the entire egg-to-adult period. However, pupae at higher temperature (44°C) increased water content similar to that at the control temperature (28°C). This demonstrated that, with when temperature increases within a certain range, pupae can adapt to higher temperature conditions by regulating water content. Taken together, this suggests that thermal fluctuation can lead to the down regulation of body water content in adult stages as a result of heat injury mitigation. Regulated water loss at high temperatures has also been reported in several other arthropod species, e.g., *Balaustium* sp. near *putmani* (Yoder et al., 2007), *Mezium affine* and *Gibbium aequinoctiale* (Yoder et al., 2009), *Corythucha ciliata* (Ju et al., 2014), *Paratarsotomus macropalpis* (Wu and Wright, 2015), and *Myzus persicae* (Ghaedi and Andrew, 2016). These findings indicate that insects may regulate their water loss rates to cool their bodies to avoid heat stress injury (Ju et al., 2014). Water excretion in insects also induces an increase in intracellular heat-protective compounds due to the lower content of water that remains *in vivo*; these mechanisms ultimately allow more insects to survive at high temperatures (Chown et al., 2011).

Insects also accumulate sugars and polyols as physiological strategies to avoid temperature impairment (Duman, 2001). The accumulation of low molecular weight sugars and polyols is one of the major mechanisms hypothesized to increase cold tolerance in overwintering insects (Wang et al., 2010). Most previous studies have focused on cold tolerance and there is a paucity of studies that have focused on the metabolomics of heat acclimation in insects (Schou et al., 2017). Sugars and polyols also help to stabilize proteins against heat denaturation. Aphids and whiteflies were the first insect taxa reported to accumulate polyols in response to high temperatures and with regards to protein stabilization, i.e., these compounds were functional in stabilizing protein structures against thermal denaturation (Back et al., 1979). Other studies also predicted that aphids accumulate polyols and sugars in response to high temperatures (Hendrix and Salvucci, 1998; Ghaedi and Andrew, 2016). In the present study, the total sugar content of *O. communa* female and male adults increased significantly compared to the control after the egg and larval stages were exposed to 40–44°C for 3 h, when the pupal and adult stages were exposed to temperatures ≤ 42°C for 3 h, and after long-term (egg-to-adult) phasic thermal stress. The total sugar content in *O. communa* female adults increased at 40°C but decreased at 44°C, while the total sugar content in *O. communa* male adults decreased significantly at any of the high temperatures. We suspect that *O. communa* is likely to accumulate sugar to avoid heat damage when eggs and larvae are exposed to short-term phasic thermal stress ≤ 44°C, when pupae and adults are exposed to short-term phasic thermal stress ≤ 42°C, or when female *O. communa* are exposed to long-term phasic thermal stress ≤ 40°C. At temperatures lower than 44°C, the eggs and larvae presumably have adequate time to repair heat-induced injury and accumulate sugars to protect themselves from further damage. After short-term exposure to 44°C and under long-term phasic thermal stress, *O. communa* adults do not have enough time to repair heat injuries and thus need to consume sugar for energy to escape heat stress. Both the glycogen and glycerol contents of female and male adults increased significantly after the different life stages were exposed to short-term phasic thermal stress or after the entire egg-to-adult period was exposed to long-term phasic thermal stress. These findings were consistent with those of several other insect species, e.g., *Aphis gossypii* and *Bemisia argentifolii* (Hendrix and Salvucci, 1998), *M. persicae* (Ghaedi and Andrew, 2016), and *Plodia interpunctella* (Kim et al., 2017) which accumulate sugars and polyols under heat stress. Similar results have been reported for aphids, whereby higher temperatures resulted in increased levels of sorbitol, a metabolite also known to protect silverleaf whitefly under high temperature conditions (Wolfe et al., 1998; Salvucci, 2000). A decrease in sucrose and an increase in fructose and sorbitol after heat treatment are consistent with sorbitol metabolism. Heat treated aphids resulted in an increase in several sugar alcohols that are known to be protective under temperature stress, including erythritol and mannitol (Michaud et al., 2008; Rangel et al., 2008), and galactitol and lyxitol. The accumulation of glycogen during the insect larval stage has been linked to increased body water content and is likely a source of metabolic water during dry conditions (Graves et al., 1992). Glycogen, a glucose polysaccharide, acts as an oxidative energy source during times of physiological stress. Because it binds up to five times its weight in bulk water, insects with increased levels of body glycogen also have higher amounts of internal water (Gray and Bradley, 2005). These results show that insects may regulate their sugars and polyols contents to protect against heat injury. Glycogen is produced and stored in fat body, which also mobilizes glucose to provide support under extreme temperatures (Pant and Jaiswal, 1982). Temperature affects the kinetic properties of enzymes and can have important roles in regulating glycogenolysis (Holden and Storey, 1993). Within a certain temperature range, *O. communa* can accumulate glycogen to survive the high temperature conditions. The significantly increased levels of metabolic parameters (water-soluble protein, triglycerides, mannitol, and sorbitol) of *C. ciliata* in response to high temperature stresses was also observed in both the laboratory and field (Ju et al., 2014). Compatible solutes, such as sugars, polyols, or free amino acids, have a range of protective properties, such as detoxification or stabilization of proteins and membranes (Yancey, 2005). This heat-resistance may provide a defense mechanism to counter thermal damage in *O. communa*.

Insect bodies contain lipids that have various functions, such as storing energy and as components in cell membranes (Takenaka et al., 2014). Many insects use lipids as an energy source and fatty acids are stored in the fat body in the form of triacylglycerol (Ghaedi and Andrew, 2016). Insects usually store energy in the form of lipids for later utilization during overwintering or diapause (Sadeghi et al., 2012). Accumulation of lipids to avoid low-temperature injury has been reported in *Agonoscena pistacia*e (Sadeghi et al., 2012), *Osmia rufa* (Dmochowska et al., 2013), *Ectomyelois ceratoniae* (Heydari and Izadi, 2014), and *Spodoptera litura* (Zhu et al., 2017). But lipid metabolism in insects under high temperature stress has seldom been reported. Under heat stress, insects may search for low-temperature microclimates to reduce thermal injury through behavioral thermoregulation (Amarasekare and Sifuentes, 2012). Flight is very metabolically demanding and the depletion of lipids following adult emergence is consistent with an upregulation of the metabolic rate (i.e., by 50–100-fold during flight muscle activity) (Canavoso et al., 2001). Rapid lipid use may be related to flight activities (Muntzer et al., 2015), and a decrease in the total lipid content after flight was reported in *Rhodnius prolixus* (Oliveira et al., 2006). In our indoor study and during field observations in midsummer, adult *O*. *communa* were observed to be more active to escape high temperatures stress through flight. Collectively, these findings and the observation of the lowest lipid content after adults were exposed to high temperatures in the present study indicate a behavioral lipid consumption of *O*. *communa*. Meanwhile energy-demanding repair and recovery processes under thermal stress require enough adenosine triphosphate (ATP) supply (Colinet et al., 2015), and which may expend lipids as an energy source. In this study, the lipid content of *O*. *communa* adults decreased significantly after the pre-adult stages or entire egg-to-adult period were exposed to short- or long-term brief thermal stress, respectively, and the stored lipids may have been used as a source of metabolic energy for physiological processes. Consistent with our findings, Ghaedi and Andrew (2016) also showed that the triacylglycerol content significantly decreased in *M. persicae* adults that were repeatedly exposed to high temperatures. These results suggest that lipids play an important role in behavioral thermoregulation and in repairing processes under thermal stress.

Sex-specific responses to heat stress in insects have been reported in numerous studies with females often having been observed to be the more stress-resistant sex (Karl et al., 2014; Esperk et al., 2016; Schou et al., 2017; Chen et al., 2018b, c). Our previous studies showed that *O. communa* females had higher survival and antioxidative enzyme activities (Chen et al., 2018b, c), which indicated a higher thermal tolerance of female *O. communa*. In the present study, higher contents of total sugars and lipids were observed in *O. communa* females after the different developmental stages (egg, larval, pupal, and adult) or entire egg-to-adult period were exposed to brief, high-temperature stress. In some insect species, females have been found to store a greater amount of energy substances (e.g., lipids) than the males for investment in egg production (Muntzer et al., 2015); however, the higher total sugar and lipid contents in *O. communa* females under stress may be a survival strategy rather than a reproductive strategy. Glycogen in insects is mainly used for trehalose and polyol synthesis (Storey and Storey, 1983), and the relatively low glycogen content found in *O. communa* female adults after the entire egg-to-adult developmental period was exposed to the high temperature treatments may have compromised the availability of carbon atoms for the biosynthesis of trehalose and polyols to improve heat tolerance. The lower water content in *O. communa* females under heat stress indicated a higher water loss rate and rapid cooling ability. Stage-specific responses to heat stress have been observed in many insects (Zhang et al., 2015; Chen et al., 2018b, c), it is generally assumed that more mobile stages require less physiological protection against heat stress because they can behaviorally thermoregulate (Blanckenhorn et al., 2014), and less mobile life stages (e.g., eggs or pupae) are expected to show increased plasticity in their responses (Rinehart et al., 2006). According to this principle, stage-specific metabolic response of *O. communa* to heat stress was also observed in the present study, and adults should accumulate more total sugar, glycogen, and glycerol contents, and less water and lipid contents after the exposure of eggs or pupae to high temperatures. However, this trend was not consistent and did not always obey the Bogert effect. It has been suggested that insects could recover before and during the adult stage or repair injuries during cooler periods between high temperatures (Zhang et al., 2015; Chen et al., 2018c; Ma et al., 2018). Different stages experience relatively abrupt physiological and morphological changes during metamorphosis, which leads to a high degree of independence for each stage (Potter et al., 2011), which may lead to different results in terms of temperature response and the Bogert effect. The present study focused on the physiological responses of subsequent adults after the exposure of different developmental stages to heat stress. However, metabolism will change immediately in treated stages in response to high temperatures, and thus, the physiological responses of treated life stages of *O. communa* to heat stress need to be studied in the future.

In summary, our results demonstrated the physiological of *O. communa* to improve its heat tolerance after the different life stages were exposed to heat stress. Both the exposure of the different developmental stages or throughout the egg-to-adult period to brief short- or long-term high temperature treatments, respectively, lead to significant changes in the contents of the physiological metabolites. The physiological responses are expected to play a fundamental role in heat-stressed *O.* co*mmuna*. Eggs were shown to be sensitive to thermal fluctuations during the brief exposures to high temperatures; however, the larvae and adults were able to thermoregulate via behavioral adjustments, e.g., move to shaded areas. Our study significantly improves our understanding of the responses of *O.* co*mmuna* to thermal stress and thus improves our ability to predict the impacts of climate warming on this important species. In addition, our findings (including previous studies, body size and weight, antioxidant responses, and life history parameters of *O.* co*mmuna* under heat stress, Chen et al., 2014, 2018b, c) indicate that even during the summertime, *O.* co*mmuna* is still expected to be an effective biocontrol agent for ragweed in central and southern China.

## Author Contributions

ZZ and FW conceived and designed the work. LY and JG helped in the theoretical analysis. HC and CZ performed the experiments. HC and GS wrote the first draft of the manuscript. All authors contributed to the manuscript revisions.

## Conflict of Interest Statement

The authors declare that the research was conducted in the absence of any commercial or financial relationships that could be construed as a potential conflict of interest.

## Footnotes

1 http://data.cma.cn/site/index.html
